# A qualitative study exploring young offenders’ perspectives on alcohol and other drug health promotion

**DOI:** 10.1186/s12889-022-12953-z

**Published:** 2022-03-22

**Authors:** Emily Deans, Jioji Ravulo, Elizabeth Conroy, Joseph Abdo

**Affiliations:** 1 Youth Solutions, Campbelltown, Australia; 2 The University of Sydney, Sydney, Australia; 3 Western Sydney University, Campbelltown, Australia; 4 University of Wollongong, Wollongong, Australia

**Keywords:** Alcohol and other drugs, Youth offenders, Prevention, Harm reduction, Health promotion

## Abstract

**Background:**

Drugs and alcohol can cause significant harm to individuals, families and communities. Young offenders represent an important population group, which often sport many characteristics that make them highly vulnerable to experiencing harm from drug use. For decades, research has shown the complexity of health behaviours and the need to consider consumer perspectives to respond and support different populations effectively.

**Methods:**

This study utilised qualitative inquiry to explore young offenders’ (aged 13 to 18 years) experiences with drug use. The study sought to discern the pathways to drug dependencies for young people and to understand how community organisations can better support young people involved with the justice system.

**Results:**

Three themes were identified in the data. First, the clear lack of knowledge about how to reduce harm from drug use among young offenders. Second, the structural and environmental influences on drug use and the need to develop personal skills and knowledge, alongside advocating for supportive environments for good health. Third, the power and hope that a youth advocate with lived experience can bring to the harm prevention and health promotion field.

**Conclusions:**

Community services have an integral role in ensuring drug and alcohol education is accessible for different youth populations. Importantly, health promotion organisations should raise awareness about the environmental influences on drug use behaviours, and work deliberately to include consumer perspectives in the design and planning of prevention and harm reduction strategies.

## Background

Responding to drug and alcohol issues is hugely complex and efforts to curb harms to individuals, families and communities are being made both within the treatment field and in the health promotion and prevention sectors [1]. Tobacco, alcohol and illicit drug use contributes significantly to increased chronic illness, injury and premature death, and remains among the leading risk factors contributing to the burden of disease in Australia [2]. Drug and alcohol problems are made even more intricate by layers of co-morbidity, for example the dual diagnosis of mental illness and problematic patterns of substance use [3–5], and is at times further complicated by a myriad of sociocultural factors that can impact upon an individual’s capacity to seek effective support [6, 7]. To apply a ‘one-size-fits-all’ approach to prevention, early intervention or treatment would assume a myopic understanding of what contributes to drug use, ignoring the many factors that can shape and influence health behaviours [8, 9]. Exploring the contexts of, and influences on, drug use for different population groups is integral if we are to respond with appropriate education strategies and treatment options for an increasingly diverse population in Australia, each with their own unique needs.

Similarly, involving ‘vulnerable’ consumer voices in the design and delivery of community services is an important means of harnessing knowledge pertinent to minority or marginalised population groups. Consumer involvement is gaining traction, although remains in its infancy, particularly within the AOD preventive field where education strategies are often ‘expert driven’ [10]. Baker and colleagues [11] describe a ‘vulnerable consumer’ as those individuals whose characteristics, or individual circumstances, interact with external environmental conditions to create a state of powerlessness in consumption situations. One such population of ‘vulnerable consumer’ is young offenders, who often have high rates of social disadvantage, poor physical and mental health, experience of childhood maltreatment and family violence, and high levels of out-of-home care (OOHC) and parental incarceration, often cooccurring alongside problematic patterns of drug use [12–14].

When compared to adult offenders, it appears that substance use is more heavily implicated in juvenile offending. The Young People in Custody Health Survey [15], which has a sexennial project cycle, found that lifetime illicit drug use remains common among young offenders, with 93% of participants reporting substance use. A significant proportion of this population had also developed problematic patterns of consumption, with 89% of those who had used crystal methamphetamine and 60% of those who had used cannabis, meeting the Severity of Dependence Scale (SDS) criteria for dependence [15]. Findings also show that up to three quarters of young offenders detained by police were intoxicated at the time of their offence and two thirds reported committing crime to obtain substances [15]. Problematic substance use has also been shown to be a strong predictor of previous incarceration and re-incarceration among young people in custody in Australia, with individuals categorised as ‘heavy drinkers’ likely to be reincarcerated within the subsequent 18 months, and young people who used cannabis post-release, two times more likely to be re-incarcerated [12].

### Setting

Youth Solutions is a youth drug and alcohol prevention service operating out of the Macarthur and Wingecarribee regions of New South Wales. Youth Solutions work with young people from a variety of backgrounds, with different levels of social advantage, and different experience and exposure to drug use. The service is local, tailored and invested in formative evaluation and qualitative research to explore the contexts within which young people use drugs and the factors influencing their consumption [16]. Like others within the preventive field [17–19], Youth Solutions believe that the more community services can learn about the contexts in which people use drugs, and the social, cultural and environmental factors which shape drug behaviours, the better positioned we are to support, care and make change within this space.

## Methods

Utilising in-depth qualitative research design, we sought to unpack the drug and alcohol priorities for young offenders aged 13 to 18 years and explore the psychosocial factors which influenced drug use among this population. As a preventive service, Youth Solutions were particularly interested in identifying the lessons learned for designing appropriate and inclusive alcohol and other drug education strategies to guide project delivery and health promotion and harm reduction work more broadly within the alcohol and other drug sector.

### Approach

This research is guided by a Constructivist Grounded Theory (CGT) epistemology, which acknowledges the co-creation of knowledge and individuals’ subjective ‘truth’ [20]. The research team and the participants in this study have a particular lens in which they see the world, which impacts upon the way data is collected, coded and interpreted. A CGT approach acknowledges that research is a social construct, mutually created by researcher and participants.

### Ethics

The team sought approval from two human research ethics committees, including Western Sydney University HREC (H12964) and the Aboriginal Health & Medical Research Council Ethics Committee (RN 1496/19). The research project was then approved by the Youth Justice Research and Evaluation Steering Committee within the NSW Government Department of Communities and Justice. All methods were carried out in accordance with the Australian Code for the Responsible Conduct of Research; guidelines jointly developed by the National Health and Medical Research Council, the Australian Research Council and Universities Australia.

### Recruitment and participation

Participants were recruited through Youth Justice Centres in New South Wales and the research team worked collaboratively with centre staff and caseworkers to identify potential participants. Centre staff helped group participants appropriately (and safely), and included young people who were stable enough to actively contribute to a focus group discussion. Participation was voluntary and all young people were given an information sheet prior to giving their written and verbal consent to participate. For young people under the age of 18, guardian consent was sought and where appropriate/possible, this included verbal consent from parents. The young people in this sample had access to counsellors, caseworkers, health clinicians and/or Occupational Therapists as needed, and were reprimanded in a facility that could provide them with access to targeted psychoeducation and referral as required. All participants received a $40 voucher as reimbursement for their time spent participating and as an acknowledgement of their strength in sharing their insights and personal experiences, which were often layered with grief, trauma and social disadvantage. Vouchers were held in participants’ stored belongings for future use when released from custody and all focus groups were catered with food.

### Data collection

The team conducted 9 semi structured focus groups with 30 young people (see Table 1 – Focus Group Composition) currently being reprimanded in a Youth Justice Centre in NSW from January 2021 to May 2021. Our approach as a research team was to privilege the young people’s views and voices. A few participants attended more than one focus group (*n* = 3), to support their peers in participating and to help facilitate a safe environment. Focus groups were audio recorded for transcription purposes. All focus groups were guided by four broad conversation starters:

1. *Tell me about your experiences with alcohol and other drugs?*
2. *What influences your drug use?*
3. *What about help services? Can you tell me about any you have accessed?*
4. *What are your thoughts on alcohol and drug education?*

**Table 1:** Focus group composition

Focus Group 1	Male unit: four participants Age: 14 years Cultural background: Aboriginal & Torres Strait Islander (*n* = 2) and Pacific Islander (*n* = 2) Drug use: alcohol, cannabis, MDMA & methamphetamine Two guards present
Focus Group 2	Female unit: four participants Age range: 16 – 18 years Cultural background: Aboriginal & Torres Strait Islander (*n* = 1) and Anglo (*n* = 3) Drug use: cannabis, cocaine, MDMA & methamphetamine Two guards present
Focus Group 3	Female unit: four participants Age range: 14 – 17 years Cultural background: Anglo (*n* = 4) Drug use: alcohol, benzodiazepines, cannabis, ketamine, MDMA, & methamphetamine Two guards present
Focus Group 4	Male unit: two participants Age: 14 years Cultural background: Aboriginal & Torres Strait Islander (*n* = 2) Drug use: cannabis and alcohol Two guards present
Focus Group 5	Mixed unit: four participants (3 males and one female) Age range: 13 – 18 years Cultural background: Undisclosed Drug use: alcohol, buprenorphine, cannabis, cocaine, LSD, MDMA & methamphetamine Two guards present
Focus Group 6	Female unit: five participants Age range: 15 – 18 years Cultural background: Anglo (*n* = 1), Aboriginal & Torres Strait Islander (*n* = 3) and Pacific (Samoan – Tongan) (*n* = 1) Drug use: benzodiazepines, cannabis, cocaine, GHB, LSD & MDMA One guard present
Focus Group 7	Female unit: two participants Age range: 16 – 18 years Cultural background: Aboriginal, South African (*n* = 1) and Anglo, Italian (*n* = 1) Drug use: benzodiazepines, buprenorphine, cannabis, GHB, GBL, heroin, ketamine, LSD, MDMA, methamphetamine One guard present
Focus Group 8	Female unit: five participants Age range: 12 – 15 years Cultural background: undisclosed Drug use: benzodiazepines, cocaine, heroin & methamphetamine *Difficult focus group which required guard intervention and presence of caseworker to support discussions Three guards present
Focus Group 9	Male unit: three participants Age: 14 years Cultural background: Aboriginal & Torres Strait Islander (*n* = 3) Drug use: alcohol, cannabis, MDMA One guard present

The research team were also accompanied by NSW Youth Justice staff, who had built rapport with participants and where needed helped to facilitate discussions with the young people.

### Data analysis and interpretation

The research team used open coding techniques to identify the thematic narratives which emerged from the data. Coding was completed by members of the research team who conducted the focus groups and who transcribed the audio files (Author One and Author Four). The project leads listened to audio recordings several times and re-read transcripts to become familiar with the data and employed the constant comparative method of data analysis [21] to identify similarities and differences in participant accounts. Author Two and Author Three assisted with the interpretation of codes and the meanings attributed to participants’ commentary.

## Results

### Participants

In total, 30 young people aged between 13 – 18 participated in the study with a proportion of the sample identifying as Aboriginal or Torres Strait Islander (*n *= 11, 37%), Pacific (*n* = 3, 10%) or from a refugee background (*n* = 1). Some participants preferred to keep their ethnicity and age unidentified. Sixty percent of the sample were female and forty percent were male. All participants had used a range of illicit substances prior to being reprimanded including alcohol, methamphetamine, cannabis (referred to as ‘yarndi’ or ‘budd’ for most participants), LSD (colloquially referred to as ‘acid’ among our sample), GHB and GBL, MDMA (referred to as ‘pingas’ among our sample) and cocaine. Participants also reported mixing illicit substances with other pharmaceutical drugs including benzodiazepines (referred to as ‘xannies’ and Valium, denoting the pharmaceutical brands of benzodiazepines) and ketamine. Fewer participants had used opioids (*n* = 3) including heroin and buprenorphine. A proportion of participants (*n* = 8, 27%) disclosed that they had sought treatment and support through rehabilitation, however this was typically mandated as part of their sentencing agreements. Participants had different custodial trajectories, and the length of time spent in the justice system was diverse.

### Thematic findings

Three themes were identified in the data that related to the lessons learned for prevention and health promotion. First, the lack of knowledge about the consequences of drug use or how to reduce harm. Second, the structural and environmental influences on drug use and the need to develop personal skills and knowledge alongside advocating for supportive environments for good health. Third, the power and hope that a youth advocate with lived experience can bring to the harm reduction and health promotion field.

#### *‘Drugs can damage you…I didn’t know that until I was in custody’*

The first theme identified in the data related to the lack of understanding around the risks associated with drug use, and the clear gap in participants’ knowledge of harm minimisation strategies. On the contrary, participants were incredibly knowledgeable about the variety of substances on the market, where to access them, and how to make money from dealing drugs. Participants had used numerous substances including benzodiazepines (‘xannies’, ‘Valium’), tranquilizers (rivotrils), anti-psychotic medication (such as ‘Seroquel’), MDMA (ecstasy, ‘pingas’), ketamine, mushrooms, lysergic acid diethylamide (LSD), cannabis (‘yarndi’, ‘pot’, ‘budd’), gamma-hydroxybutyrate (GHB), gamma-butyrolactone (GBL), cigarettes and alcohol. Poly drug use was common and participants drug use was largely driven by contextual and situational factors (which will be explored in more depth in theme two). All participants in this research study described being introduced to drugs at an early age (between 7 and 14 years old), and despite having easy access to substances through familial and peer networks, many simply did not know about the risks associated with drug use: *“I used to think it’s a good thing! My brother used to be on it all the time, he used to say he loves it, like he was addicted to it, so I thought it was a good thing”* (Female, 18 yrs, Anglo). One male participant (14 years, Aboriginal) reflecting upon his journey of cannabis use, said: *“I didn’t really know what it really was…if someone taught me before I had it (at age 12), I probably wouldn’t be on it… I probably wouldn’t be in here”.* Another young male (14 yrs, ethnicity undisclosed), when asked whether he knew that alcohol effects the liver negatively, exclaimed: *‘No! I just wanted to have a good time.’* The following participant who started using methamphetamine when she was 11 years old and who recently started using gamma-hydroxybutyrate (GHB), described how drug use helped her escape to a world where anything was possible, and that ‘harm’ and ‘risk’ weren’t part of the picture until she witnessed how things could go wrong.

*“When I’m on it, I feel like, you know, I’m in this fantasy… I didn’t realise it’s (GHB) another drug for like, men to give girls, to like, you know, put them to sleep and rape them. I didn’t know that it was dangerous until I seen it with my own eyes, almost happened to me. I didn’t even know, if that happened to me, who knows? I’m gee’d out, gee’d out heaps of times and I’ve been around a lot of men in a room… I’ve seen girls get raped in front of me, I’ve seen them get gee’d out and shit, it’s dangerous.”* (Female, 18 years, Aboriginal)

A lack of awareness about the risks of drug use continued for some participants, despite having experiences with alcohol and other drug treatment. For example, after having traversed a system that was in place to support and treat cannabis and ecstasy dependency, the following young person had little knowledge about the risks associated with methamphetamine use. She described the effects of losing weight, gaining motivation and confidence as drivers of continued ice use after being introduced to the drug through a close friend.

*“I was 13 and I just gotten out of rehab for pot and caps, and then me ex, well he wasn’t me ex at the time… gave me it for my first time because we tried everything together. I was like ‘oh yeah, fuck yeah, what is this?’. He gave it to me and I was like ‘I don’t know how to light it’… but then I didn’t know… like it was going to be… the satisfaction as well, like, you see it….’wooooossshhhhhhh’. After it you are like ‘fucking what cunt?’… I just wanted to try it and then it became an addiction.”* (16 years old, Aboriginal)

The longer the drug use trajectory, the more ‘tea-house’ networks participants appeared to have, and the more likely participants had experienced risky situations related to their drug use. Contrary to some assumptions that perhaps could be made, the young people involved in this study were not ‘anti-prevention’, and instead saw the value in education strategies, as most of the participants had experienced some level of harm from their drug use. This harm manifested itself in numerous ways, to being reprimanded for armed robberies while high on methamphetamine, to damaged and strained family and friend relationships, to experiencing the health consequences and physical changes due to frequent drug use: *“I started my first crime when I was 11. I’ve been through it all, smoked every drug in the book and at the end of it, it’s all dangerous, you’re face, you age, you’re ugly…you can’t stop unless you hit rock bottom”* (Female, 16 years, Aboriginal).

Awareness and knowledge about how to decrease risk of harm from drug use was limited and focus group discussions often dissipated when participants were asked specifically about harm mitigation strategies. There were three exceptions, with a 17-year-old female explaining the dangers of mixing drugs, a 14-year-old male describing dangerous settings for drug use, and a 14-year-old female who explained the importance of hydration and checking on friends’ consciousness and breathing after drug use.

*“If I see people on the xanys, I’ll tell them don’t drink because that’s when people get hurt aye…when they mix alcohol and xanys, then you’re really like out of it, especially if you are a little person, like a skinny little girl, you can’t handle it, your body weight can’t handle it but you think you can, but you can’t. If you are little and your like when other girls that are maybe bigger than you or boys… and like, matching what they’re taken, you’re going to be in a different state to them.”* (Female, 17 years, undisclosed ethnicity)

The second example of a harm reduction scenario playing out was in relation to cannabis use and the setting in which it was taken. The following 14-year-old Aboriginal male, who had lived the struggles of having parents who had developed problems with methamphetamine and alcohol, described how he had assessed a situation and made the call to stay sober to protect his friends from harm.

*“There was one time, um, I wasn’t smoking at the time, and we were at the car park, very high, and then there’s a fence and we jumped over the fence, and then there was a ledge. And then if you fall off the ledge, it’s like 60 feet down, and if you fall off you die. My two mates they were smoking, they were high, and they were sitting on the ledge and they were leaning off it. They were high, they could of fell off, and if they fell out they would of died, it was like 60 foot. Yeah it’s dangerous”*

Interviewer: Were you worried about them?

*“Yes, that’s why I didn’t get high, cause you know, the ledge”*

Interviewer: Yeah, so you assessed the situation, you were like ‘it’s not safe to have marijuana so I’m not going to smoke it here because it’s dangerous?’

*“Yeah.” *(Male, 14 years, Aboriginal)

There was a general consensus across focus groups that educational workshops delivered in school-based settings failed to engage with some of the most vulnerable populations with many acknowledging that they had started using drugs at an early age and were frequently absent from environments that could have provided educational and harm reduction support: *“I started to get into drugs when I was around 12” … “yeah and I hardly went to school, even in primary” … “I missed about four years of school! I missed 6, 7, 8, 9” … “yeah I went to year 6.”* For those who were able to receive some form of drug education during high school, it was clear that the messages were not tailored to their needs, and that knowledge around harm reduction strategies was missing from the program outcomes. Those who had started using drugs before high school, exclaimed that they were *‘already fucked up’*, *‘already stuffed because I’m on it’* and the default when learning about the consequences of drugs appeared to be a position of helplessness. The following quote from a 16-year-old female points to the way in which some young people disregarded prevention messages as they were tailored to young people who didn’t use drugs.

*“I was reading stuff (drug education) like that on Facebook and scrolling through like Instagram and that, and stuff about drugs come up, my fault, I’m already using them, no point stopping now.”* (Female, 16 years, Anglo)

#### *‘I do yarndi to take away my stress’*: Environmental drivers of drug use behaviour

The second theme identified in the data related to the contextual influences on drug use and pointed to the importance of addressing psychosocial needs over and above an individual’s AOD knowledge and skills in harm reduction. Participants’ lived realities revealed the complexities of making a real influence through education alone given the way substance use was entangled in participants’ social networks and relied upon to manage mental health issues and stressful life experiences. Drug use was often embedded in social and familial networks, with many participants experiencing trauma, mental illness, and episodes of intense grief from losing uncles and parents, all before the age of 18 years. Some of the experiences that were shared included stories about watching loved ones die from a drug overdose, losing family members in alcohol-related car accidents and working through strained and abusive relationships (and for some a complete lack of social connection and experiences of rejection and isolation). Mental health issues were common, and for some, the primary motivating factor for the uptake of drugs. The following participant described a helpless situation, where using drugs seemed like a ‘logical’ fix to her problems.

*“I started drugs because I had fucking nothing, or I think of myself as fucking…. I was real down and I wasn’t happy, I had ADHD and all this shit. I was like real hypo and fucking all this bro. I just wanted to be relaxed and a kick back kind of person. So, I started using yarndi…and then I was like ‘fuck yarndi cuz, let’s get on the crack.’”* (Female, 18 years, undisclosed ethnicity)

Another young female participant (16 years, Aboriginal), described how mental health problems and lack of awareness around where to access support and resources had led her down a path of problematic drug use. She explained:

*“On the outside, I lived in a refuge away from my family. I was quite depressed and the only way I could feel happy is if I took drugs. I was addicted to the high and it was very expensive as well. So I couldn’t afford it, so I’ll do crime to get the drug”*

Interviewer: Do you want to talk me through how you were feeling in that space before you decided to use drugs?

*“Scared, didn’t know what to do. Didn’t know how to help myself. Yeah, on drugs I felt like I didn’t have to do anything, or that it didn’t matter if I made a change or not. Didn’t matter if I was sad, nothing mattered at all. I felt like that (using drugs) was my only option… but there is supports that can help you if you feel like that, I know that now.”*

Positive role models were clearly a protective factor (and a strong motivator for behaviour change) as the following female participant explained: *‘I’ve been off the drugs for five months… I met this guy and he’s copped a lot of shit…and um no one really looked after me and him being by my side and showing me that he cares… someone there…made me want to make a change in my life. Cause some of us grow up with no one at all. He didn’t leave like the way my family left’* (16 years, Aboriginal). Another young male (14 years, Pacific) described having a cousin who was an elite sportsperson who didn’t use drugs and expressed his desire to discontinue his drug use to achieve his own fitness goals. Many participants however, lacked positive and ‘healthy’ social connections. The following participant, who primarily used GHB, described the dominating figures in her life that had led her down a pathway into custody:

*“I hang out with 40-year-olds, I hang out with 30-year-olds, even 50-year-olds. They still talk about ‘oh back in the day’…they’re not good role models you know? Like, you might think, they like care about you, they don’t care about you. They just there to feed off you. They want you to do the crime so you can go in and do minor, cause we’re juveniles, you know? They want you to do the crime because they know that you’re only going to get the minimum time but if they do it, they’ll get years. That’s what I know.”* (Female, 15 yrs, Samoan-Tongan)

This experience was similar to another participant (female, 14 years, Anglo), who described being introduced to methamphetamine at age 14 at a 40-year old’s house, expressing *“it’s normal for me”*. Another young female (15 years, Anglo) described that her first experience with using ‘xannies’ (unprescribed) was when she was 14 years old and her boyfriend spiked her drink. From then on, Xanax became a ‘usual’ add on to partying with her partner and his friends. The following excerpt suggests how simple harm reduction strategies like having a sober friend were unheeded, which meant that she was at significant risk of harm from drug use, particularly given the contexts in which she used.

*“Once I was on xannies and alcohol and I blacked out. I don’t remember what happened but like I was on the back of this guy’s shoulder because I couldn’t walk. And, like, um, like, he was behind some of my other friends. My mates were walking ahead, and then, they turned around five minutes later and he was just like gone with me on his shoulders. I don’t know where the fuck he took me and then apparently, he was gone for 30 minutes and then he apparently came back without me, because the cops took me. He dumped me on the side of the road, on the side of the highway. Then someone called the ambulance”*

Interviewer: A friend?

*“Nah”*

Interviewer: Just someone who passed you by?

*“Yeah.”*

For some, the institutional setting of being reprimanded was an introduction to help and support via NSW Justice staff that cared for the wellbeing of the young person, and through case management, a chance to start again in an environment that had structure, support and routine. This point was reinforced and made clear by comments from one of the Youth Officers (focus group 8). He said:

*“I’ve worked with (young female) now for a little bit. Aye sis? We’ve been together and she’s one of the greatest girls that have ever come through this place. When she comes through and she’s been on the drugs, and the other two girls, they come through having a hard time adjusting at first and the girls that are sitting in front of you today are beautiful women. They are really strong and it’s amazing to see them talk. So it doesn’t matter what trauma, what they’ve been through or why they lost themselves, for them to be able to talk about this today and really help with that. It makes me really proud of them.”*

For some of the participants in this study, carceral spaces was a safer place to be, and meant that you ‘no longer had to look over your shoulder’. When asked *‘how do you feel about being in here (prison) and not being able to use drugs?’,* an 18-year-old Aboriginal female responded *“Look it’s a good thing, I was going down a sad path. So I’m kind of happy, not happy I’m in here, but I’ve been off the drug for three weeks now’.* A group of four male participants (14 yrs, Aboriginal, Pacific) described that prison was a place of support, a place to get help, a remedy and catalyst for change. When asked the following question: ‘*do you know any alcohol and other drug services that can help people with drug problems?’*, the young males replied in unison; *“Jail”.* For others, support through AOD issues came from fellow inmates, as shown by the following quote from the same Youth Officer:

*“So what (young female) is really good at is when girls come down and they’re abandoned and they’re on their come down, so they’ll come in pretty scattered. (Young female) can actually pick up on (the) situation and spend time on getting them to come to basketball. I don’t know if she realises that she does it but a lot of times in the unit the girls sneak under the doors while they’re in the unit. They will talk to girls…they’ll have a laugh… a lot of positive energy.”*

#### *‘It’s way easier said than done’*: the importance of a lived experience

The third theme identified in the data related to the power of incorporating lived experience within the alcohol and other drug prevention, harm reduction and treatment sectors. A lived experience provided hope for the young people in this study who were experiencing harm from their drug use, and for some (not all), navigating the justice system specifically for drug-related crime. Some participants explained numerous reservations about seeking help from others (including both services and peers) and that building rapport was fundamental in encouraging them to access treatment: *“Everyone thinks they know what is best for you, but they really don’t. Oh my God ‘I understand’ – that gets on my nerves.”* It seemed that services who didn’t have a lived experience arm risked perpetuating the ‘individual responsibility rhetoric’ and were more likely to be positioned as implementing scare tactics to influence behaviours without understanding or acknowledging the complexity of drug behaviours and the risk decisions that individuals have had to grapple with. Embedding lived experiences was perceived as a helpful strategy to conceptualise alcohol and other drug problems through a structural lens. Service providers that had experienced social disadvantages and difficult drug trajectories, were praised for providing the most valuable support for the young people we spoke to. The following excerpt from focus group 3 illustrates this point:

*P2: “The lady that runs it, she’s cool, you can relate to her cause she used to be on the street and stuff. She’s a relatable person and helps out all the kids from the community... she’s always feeding the kids... ‘I’ve got no food’…if you’ve (been) suspended from school…. you don’t want to stay home cause like heaps of kids in my area get suspended, when they stay at home, their dads are home drunk and they get bashed. So she lets you chill out at the centre… it touches you way when people come in and they’ve gone to jail and ‘yeah I did drugs but look at me now’, that’s when it hits you hard and maybe like ‘wooahh, maybe I can stop’”*

*P4: “Because they know what it’s like, and I think for people that haven’t done drugs, it’s way easier said than done”*

*P1: “Like people that haven’t done they can be like ‘oh yeah, get off the drugs. It’s bad for you’, people that have done it, experienced it, they can be like ‘yeah, I know it’s hard but you can push through it and do all these difficult things, you know, stop it’”*

*P2: “And it’s good to know, like, what everyone used to get through it and what was the hardest thing for other people, so you know what obstacles you’re gonna face, yeah, get people who have been through it, people that fought through it, who have, like, experience with it.” (Focus Group 3, 4 females between the ages of 14 – 17 years, Anglo)*

Further to these points, there was a willingness to give back within the alcohol and other drug field and prevent other young people from walking the same path as the participants in this study. This was a finding that we believed was particularly powerful; an exceptional resource that could be used within the preventive field. As we discussed what alcohol and other drug education could look like, participants were passionate about using their own voices to influence change and acknowledged that it would have been *‘better to be aware of what you’re getting yourself into’.* The following excerpt from focus group 6 (5 females aged 15 – 16 years, Anglo, Samoan-Tongan, Aboriginal) illustrates these points:

*P3: “I’d love to go teach kids about the effects of it, what it does to you, how it can harm you. And like, I don’t know, it could, like some drugs cause death. I know that for a fact – overdose! I’d love to do that, oh my god”*

*P1: “Yeah, I’d love to do it too”*

*P2: “I think it only really means something if it comes from who, like, had a lived experience”*

*P1: “Yeah, true, true”*

Many participants were fervent about wanting to protect other young people from drug dealers ‘hooking’ them up and preying on their vulnerabilities (including for example mental illness and lack of confidence). Participants were asked what they would say to a young person wanting to use drugs, and responses overwhelmingly focused on the risks of developmental problems and social consequences (like for example going to jail and missing out on future opportunities, which were learned through experience): *“You get locked up, sometimes you get people bashed and robbed, and yeah it’s absolute rubbish”* [Male, 14 years, Aboriginal]. Below is an excerpt from focus group 3 which was facilitated with four female participants aged between 14 and 17 (Anglo), which further illustrate these points:

*P3: “I don’t want to be smoking ice around them (my friends), especially they will, like, maybe get on it. I wasn’t going to be the person to introduce it to them….”*

Interviewer: So you felt responsible too in a way, when you were on it? ‘I don’t want to smoke around these people because they don’t use it’

*P3: “Yeah, and like if I’m at a tea house and a little twelvie comes around…(clicks fingers), I’m like ‘get out now’, like, a little kid comes tryna get on the pipe, I’m like ‘go home’ I always stick up for the little kids”*

*P2: “Yeah I’ll rob dealers if I know they sell to little kids, because fuck em, they sell to little kids, lets go get them. Like I feel justified, you know, like fuck them they sell to little kids”*

Interviewer: Have you seen that? People giving young 10-year-olds weed?

*P1: “Yes! Just because they’re making money, they don’t care”*

*P2: “Yeah, they don’t care, a dollar is a dollar to them, they don’t care where it comes from”*

*P3: “Yes some people don’t care.”*

Participants described how they would use their own experiences as cautionary tales to prevent others from living a life dictated by drug addiction, and to avoid experiencing ‘filthy’ spaces, and physical consequences of memory loss and delayed learning: *“My memory was fucked. I don’t remember shit. Like everyone messages me on snapchat and five minutes later I’ll be like ‘what the fuck are we talking about?' Like when I’m talking, like when I’m driving as well, I know what I’m saying but it will take me forever to finish saying it. I hate it*” (Female, 16 years, Aboriginal, South African). The following 18-year-old female (Italian), who had grieved the loss of her dad at a young age, and who had settled into drug dealing to make money to survive said:

*“I’ll show them me. You want to end up like me bro? Give me the drugs! Nah I’m actually, I may be like a bad, like, I’m bad on the drugs but I’m a good influence when it comes to other people. I hate seeing little kids on drugs and that. Fucking makes me upset. The shit I’ve been through, I’ll never wish it on no one, never wish it on anyone strait out. I hate doing it myself.”*

## Discussion

This study investigated the factors which shape young offenders drug use and sought to map the lessons learnt for implementing effective health promotion strategies. The findings raise three important points to consider further.

First, while the need for universally targeted, settings-based health promotion work is important [22], our findings suggest that this approach within the AOD sector fails to reach some of the most disadvantaged and vulnerable young people. Universal approaches (while attractive to funding bodies and amenable to systemised and controlled evaluation) may not be well positioned to meet the unique needs of distinct population groups; population groups who must have a seat at the table in identifying health problems and how to respond to them [23, 24]. We support a Freirian approach as described by Wallerstein and Bernstein [23] and recognise that ‘knowledge’ should emerge from community groups sharing experiences to understand the social influences that affect their health [23]. Importantly, the conversation must be authentic, iterative, and ongoing [16].

Embedding drug and alcohol education pathways in school-based and mainstream institutional settings alone, may do very little to support the young people who live in environments where drug use and dealing is normalised and where the uptake of drugs is a by-product of survival and trauma [25]. Broadening the settings through which appropriate drug education resources can be accessed, for example, through alternative learning environments such as non-denominational and independent secondary schools, church and youth groups (which were explicitly identified by the Pacific young people in this study), neighbourhood/community centres, public libraries, and homelessness hubs, could be one way to improve health equity. Such diversity would enable young people across different settings and from different levels of socio-economic advantage access to education about drugs and alcohol to make an informed health choice. Importantly, prevention and health promotion strategies can be informal, flexible and creative; to challenge the status quo and typical design process of intervention programs, particularly those targeting vulnerable youth populations, is important. The participants in this study taught our team that providing a safe space for young people to talk openly about their drug attitudes and behaviours, in an environment that was supported by non-judgemental health promotion staff (who had an appreciation of harm reduction and risk mitigation as opposed to abstinent approaches), served to increase and share preventive knowledge amongst participants. Work by Duke and colleagues [26] also supports this notion and suggests that creating a safe space to build AOD harm reduction knowledge is integral to effective interventions, and that young people place value on caring, trusting and building relationships with non-judgemental role models, rather than the program content itself. This point further reinforces the need for targeted, local and participatory health promotion strategies, and the opportunity to enhance efforts within existing health service infrastructure, including rehabilitation and treatment, as outlined in the NSW Ministry of Health ‘Strategic Prioritisation Framework for AOD Research and Evaluation’ [27].

The second point which warrants further discussion is the need for health promotion strategies to move beyond ‘individual responsibility’ to creating supportive environments for good health (for all). This can start with an acknowledgement of the way social institutions and environmental structures effect health behaviour, and can move towards activist work within the health promotion and preventive field. The argument that social, physical and cultural aspects of an environment can have a cumulative effect on risk decisions and health behaviour isn’t novel [28–30]. However, the calls for health promotion to better acknowledge and incorporate multi-level interventions (rather than focusing solely on the individual and their knowledge) have gone largely unheeded [19]. Merton’s [31] work on ‘anomie theory’ points to the ways in which social structures may ‘exert a definite pressure’ upon individuals to engage in ‘non-conformist’ and perhaps ‘risky’ behaviours, and is an important sociological perspective to draw upon to understand risk decision making. Merton [31] explains that individuals operate in a society that place a heavy emphasis on specific symbols of success (often unduly exalted), for example, socio-economic advantage. However, there exists significant class differentials in accessing these types of success symbols, which Merton [31] suggests may eventuate in ‘illicit’ attempts to acquire them. Perhaps a calculated response to the resources and opportunities afforded. Many of the young people in this study made decisions to use substances, in the context of family and social environments where drug use was normalised and accessible, to acquire ‘success symbols’, for example, an opportunity to make money, or to cope and manage the effects of trauma. Health promotion strategies that focus solely on the motivations and perceptions of the individual, and which disregard the social settings, environments and policies that hinder the uptake and maintenance of ‘healthy’ behaviours, are unsustainable [32] and we risk widening the equity gap between privileged, well-resourced populations and marginalised groups [28]. Some scholars describe ‘education’ as ‘liberation’, a type of social action to promote participation of individuals, families, communities and organisations in gaining control over one’s life and society [23]. We would argue that advocacy work has an important place within health promotion activities, and that community organisations can take small, measured steps to address the contextual factors that influence drug and alcohol behaviours. This may involve distributing drug education resources and upskilling those who support vulnerable young people (youth justice staff, case workers, parents, carers, community workers), building new partnerships to support multidisciplinary health promotion teams, and building awareness around the influence of environmental factors on health among stakeholder groups, community leaders and funding bodies [19].

Finally, a deliberate effort to embed participatory approaches in health promotion strategies to identify the problem from the perspective of ‘the consumer’ is paramount. Sociological theorists have worked to explore how knowledge about a problem or issue (eg drug use) is created and reproduced within society [33]. For example, Thom and MacGregor [33] draw upon the work of various scholars to unpack the significance of ‘frames and framing’, and we believe these concepts have important implications for the way in which communities and societies consume, dissect and reproduce AOD harm prevention knowledge. ‘Framing’ is a process whereby a particular way of knowing or understanding can emerge, develop, fade, alter and re-emerge [33]. A ‘frame’ is more static, a state of knowing; helpful sign posts to help make sense of a phenomena [33]. Frames are often shared among social lines and population subgroups, they are widespread in a society, serving to draw boundaries around the acceptable and unacceptable, the included and the excluded [33, 34]. Faming can occur at both individual (micro) and societal levels (meso or macro) and is a critical activity in the construction of social reality; shaping individual perspectives which have the potential to influence policy and practice [33]. It’s important to note that individuals are active agents who may have the capacity to manoeuvre and resist framing discourse, however, an individual’s capacity to do this will be determined by social, economic, and cultural opportunities [34]. Lived experience advocates and peer workers have an important place in framing drug discourse in our society. Without a lived experience arm or ‘influence’, health promotion and harm reduction messages may be less impactful, or worse, negatively received. Young people in this study described lived experience as a tool to create hope and an important motivator for behaviour change. The value of incorporating lived experience, and involving the ‘vulnerable consumer’ in health promotion is also reported on in the literature more broadly [25, 26] and increasingly, a good practice guideline of many health promotion authorities in Australia [35, 36]. Our findings suggest that lived experiences may work to de-stigmatise harmful drug use and diminish feelings of isolation and helplessness for young people experiencing drug dependency and other harms from substance use. McLeroy and colleagues [28] view that by involving the target population in the description of the problem and its sources, important health promotion work has already occurred. Our research team resonates with this point, whereby health promotion was enacted by the young people in this study, in a space that validated their own experiences with drug use and allowed them to speak freely.

## Conclusion

Drug issues are complex, and the pathway to prevent and reduce harm, interlaced and multidirectional. This study sought to provide a platform for vulnerable young people to voice their thoughts and share their experiences with drug use to help community organisations better support vulnerable youth populations. The findings from this study reveal that some young people sit outside the mainstream institutions which typically provide support and education about drugs and alcohol. Further work is needed to ensure there is an equitable access to these resources, including harm reduction messages. Incorporating a public health framework into these resources is important in addressing the environmental factors which significantly shape drug use behaviours. Encouragingly so, co-design efforts continue to gain traction, and are likely to support effective health promotion measures, highlighting the importance of locally driven and targeted health promotion strategies.

## Limitations

Young people are a heterogenous group and themes presented here are not representative of all young offender’s perspectives or experiences. We also acknowledge that the mandatory requirement of having guards present could impact upon what young people chose to share and how they interacted with research staff. For the most part, guards were passive and did not contribute or participate in focus group discussions. There was only one focus group were guards played a more active role in discussions and this was to support research staff. We found that when guards offered their perspectives, the young people in this study were quick to challenge their perspectives and remained firm in their positions. However, we acknowledge that the presence of justice staff may stifle some young people’s autonomy and their willingness to be transparent.

Further, while our sample was diverse, the influence of cultural factors on participants relationship with prevention and health promotion programs was not explored to the point of data saturation. We would encourage other researchers working with young offenders to explore this concept further.

## Data Availability

The datasets generated and/or analysed during the current study are not publicly available due to confidentiality agreements made with research participants but are available from the corresponding author on reasonable request.

## Abbreviations

AOD: Alcohol and Other Drugs
CGT: Constructivist Grounded Theory
GHB: Gamma-Hydroxybutyrate
GBL: Gamma-Butyrolactone
LSD: Lysergic acid diethylamide
MDMA: Methyl​enedioxy​methamphetamine
OOHC: Out-of-home care
SDS: Severity of Dependence
